# Intercalary meristems revisited: from anatomical regions to cell-resolved fate systems

**DOI:** 10.48130/forres-0026-0012

**Published:** 2026-03-26

**Authors:** Shihui Niu

**Affiliations:** State Key Laboratory of Efficient Production of Forest Resources, National Engineering Research Center of Tree Breeding and Ecological Restoration, College of Biological Sciences and Technology, Beijing Forestry University, Beijing 100083, China

**Keywords:** Intercalary meristem, Bamboo, Single-nucleus RNA sequencing, Spatial transcriptomics, Cell fate, Internode elongation

Intercalary meristems (IcMs) are key drivers of internode elongation in the Poaceae and play a central role in shaping plant architecture, biomass accumulation, and mechanical strength. Despite their long-recognized biological and agronomic importance, IcMs have remained among the least clearly defined meristematic systems in plants. Historically, IcMs have been characterized primarily as anatomically inferred growth regions, defined by clusters of short columnar cells scattered throughout the nodal region, rather than as organized cellular systems^[1]^. This region-based perspective has limited the ability to resolve IcM cell identity, spatial organization, and developmental trajectories, which has left fundamental questions about IcM function and regulation unresolved for decades.

Recent advances in single-cell and spatial transcriptomic technologies have transformed plant developmental biology by enabling direct interrogation of cellular heterogeneity and spatial gene expression patterns^[2]^. However, applying these approaches to IcMs has proven particularly challenging. IcM cells are short-lived, deeply embedded within differentiated tissues, and rapidly transition from proliferation to elongation, rendering bulk transcriptomic analyses insufficient for resolving these features^[3]^. Overcoming these limitations requires approaches capable of resolving both cellular identity and spatial context at high resolution.

In this context, the recent study by Qin et al., published as a cover article in PNAS, represents a significant conceptual advance by demonstrating that IcMs can be resolved into discrete, spatially anchored cell populations with defined molecular identities and developmental trajectories. This work reframes IcMs from anatomically inferred growth regions to molecularly defined cellular systems, providing a new conceptual basis for understanding IcM organization and function^[4]^. By integrating single-nucleus RNA sequencing, spatial transcriptomics, high-resolution anatomical atlases, and functional validation, the authors establish a comprehensive framework that links transcriptional state, spatial localization, and developmental progression. Rather than treating IcMs as morphologically inferred regions, this work explicitly defines IcM-associated cell populations based on convergent molecular and anatomical evidence. These include highly proliferative short columnar cells and the stem-like IcM1 subpopulation. The reconstruction of developmental trajectories reveals that IcMs comprise multiple subpopulations with distinct fate biases, while functional validation of the *WOX2* homolog *clrGene008562* establishes a mechanistic link between conserved stem cell regulators and IcM-specific functions.

The choice of Moso bamboo is particularly strategic. Unlike rice and maize, where IcM activity is transient and spatially restricted, Moso bamboo exhibits extraordinary daily growth rates (up to 114.5 cm), an overall rapid growth stage spanning 45−60 d, and large culm diameters (8 to 20 cm) that facilitate precise tissue sampling and multiomics profiling. These attributes enable systematic dissection of developmental transitions that would be technically unfeasible in other systems.

Together, these findings motivate a broader conceptual redefinition of intercalary meristems. IcMs should be viewed not as anatomically defined growth regions but as spatially embedded, cell-resolved fate systems, in which discrete cell populations are organized along developmental continua and dynamically regulated over time. This shift parallels recent advances in bamboo genomics that have transformed Moso bamboo into a genomically tractable system. The availability of a chromosome-level reference genome has provided an essential foundation for accurate transcriptomic mapping and integrative analyses^[5]^, enabling cell-resolved developmental studies, such as that of Qin et al. These genomic resources form the foundation for cell-resolved developmental studies such as that of Qin et al. enable comparative analyses across developmental stages and species.

Despite these advances, important challenges remain. Critical questions include the following: Do other grasses possess analogous stem-like IcM subpopulations? How do hormonal gradients translate across species with divergent IcM architectures? Can lineage tracing technologies validate the predicted developmental trajectories? It is worth noting that while Qin et al. identified IcM characteristics in the basal 0.5 cm of the internode, traditional sampling methods often preserve the node-internode transition zone to avoid tissue loss. Whether more primitive or distinct IcM subpopulations reside in this transition zone remains an open question for future validation. Future studies integrating comparative genomics, lineage tracing, and targeted perturbations will be essential to assess the universality and evolutionary origins of cell-resolved IcM organization. Additionally, extending this framework to investigate how environmental signals modulate IcM activity at single-cell resolution represents a promising avenue for understanding phenotypic plasticity in grass architecture.

Ultimately, the transition from anatomical descriptions to cell-resolved fate systems marks a clear paradigm shift in IcM research. This conceptual advance establishes a foundation for future studies of internode elongation and provides a framework for dissecting other elusive, transient meristematic systems in plants. By demonstrating that spatially embedded, transcriptionally dynamic cell populations can be systematically resolved even in deeply embedded meristematic zones, this work sets a methodological standard for future investigations across diverse plant systems.

## Data Availability

Data sharing is not applicable to this commentary as no datasets were generated or analyzed during the current study.
